# A* Drosophila* Model for Screening Antiobesity Agents

**DOI:** 10.1155/2016/6293163

**Published:** 2016-05-09

**Authors:** Tran Thanh Men, Duong Ngoc Van Thanh, Masamitsu Yamaguchi, Takayoshi Suzuki, Gen Hattori, Masayuki Arii, Nguyen Tien Huy, Kaeko Kamei

**Affiliations:** ^1^Department of Biomolecular Engineering, Kyoto Institute of Technology, Kyoto 606-8585, Japan; ^2^Applied Biology, Kyoto Institute of Technology, Kyoto 606-8585, Japan; ^3^Graduate School of Medical Science, Kyoto Prefectural University of Medicine, Kyoto 606-0823, Japan; ^4^Designer Foods Co. Ltd., Nagoya 464-0858, Japan; ^5^Department of Clinical Product Development, Institute of Tropical Medicine (NEKKEN), Nagasaki University, Nagasaki 852-8523, Japan

## Abstract

Although triacylglycerol, the major component for lipid storage, is essential for normal physiology, its excessive accumulation causes obesity in adipose tissue and is associated with organ dysfunction in nonadipose tissue. Here, we focused on the* Drosophila* model to develop therapeutics for preventing obesity. The brummer (*bmm*) gene in* Drosophila melanogaster* is known to be homologous with human adipocyte triglyceride lipase, which is related to the regulation of lipid storage. We established a* Drosophila* model for monitoring* bmm* expression by introducing the green fluorescent protein (*GFP*) gene as a downstream reporter of the* bmm* promoter. The third-instar larvae of* Drosophila* showed the GFP signal in all tissues observed and specifically in the salivary gland nucleus. To confirm the relationship between* bmm* expression and obesity, the effect of oral administration of glucose diets on* bmm* promoter activity was analyzed. The* Drosophila* flies given high-glucose diets showed higher lipid contents, indicating the obesity phenotype; this was suggested by a weaker intensity of the GFP signal as well as reduced* bmm* mRNA expression. These results demonstrated that the transgenic* Drosophila* model established in this study is useful for screening antiobesity agents. We also report the effects of oral administration of histone deacetylase inhibitors and some vegetables on the* bmm* promoter activity.

## 1. Introduction

Obesity is a complex disorder, involving an abnormal or excessive fat accumulation that presents a risk to human health. It is the main cause of the cluster of metabolic diseases such as insulin resistance, atherosclerosis, and cancer, all of which can lead to the premature death of patients [1]. Obesity usually results from a combination of factors, the major ones of which are an unhealthy diet and physical inactivity. In addition, genetics play an important role in how an individual's body converts and burns energy. Heritability of obesity is related to not only monogene but also multigene [2, 3]. The recent investigations elucidate that the heritability of obesity tends to be high compared to other complex, polygenic diseases such as schizophrenia and autism. Additionally, its heritability is significantly higher than that for other complex traits such as hypertension and depression [4]. However, obesity-causing genes are complex and not yet fully understood. In order to study the metabolic syndrome,* Drosophila melanogaster* might be the evaluable nominee because it shares most of the same basic metabolic functions with vertebrates. Many analogous organ systems in humans that direct the uptake, storage, and metabolism of nutrients are found in fruit flies [5]. Moreover, the rapid growth of flies, their inexpensive breeding costs, and their small genome size facilitate screening for therapeutics or preventive agents of obesity.

The primary sites of fat storage in cells are the lipid droplets (LDs), which are organelles with a phospholipid monolayer membrane coated by numerous proteins that surround a lipid core [6]. Recently, a gene homolog of human adipocyte triglyceride lipase (ATGL) was discovered in* Drosophila* as a controller of lipid storage, namely, brummer (*bmm*). The* bmm* gene encodes LD-associated triacylglycerol (TG) lipase, which controls the systemic TG levels of flies in a dose-dependent manner. Mutation of the* bmm* gene was reported to induce obesity in flies [7].

Previously, BODIPY (4,4-difluoro-4-bora-3a,4a-diaza-s-indacene) and Nile red (9-diethylamino-5-benzo[*α*]phenoxazinone) were used to visualize intracellular fats in* D. melanogaster* [8, 9]. However, Nile red was reported to label lysosome-related organelles (LRO) instead of fat-storing LDs. Similarly, under the same conditions, BODIPY stained LRO strongly but stained LDs weakly [10]. These discoveries are increasing concerns about the results obtained from vital staining methods, which may not reflect the real* in vivo* situation. Therefore, the combination of LD staining with biochemical quantitation of TG is needed to evaluate fat storage in a body [9, 11]. Green fluorescent protein- (GFP-) tagged markers have been broadly applied to the analysis of* D. melanogaster* to reveal the localization of LD-associated proteins, such as hormone-sensitive lipase, lipid storage droplets 1 and 2, and BMM [7, 8]. GFP was also used as a fat indicator to study new fat storage regulators in* Caenorhabditis elegans* [12]. However, these studies revealed difficulties in achieving easy and rapid screening for antiobesity drug candidates, since so many LDs are contained in a cell.

In this study, we introduced the* bmm* promoter fused with the* GFP* gene into* Drosophila* to reveal whether the transgenic fly could be used as a lipid storage indicator and serve as a marker for the effective screening of antiobesity agents. Because GFP contains a nuclear localization sequence, its signal is expected to be easily detected in the nucleus of the* Drosophila* salivary gland, which is very large owing to endoreplication. Therefore, we revealed the relationship between lipid accumulation and* bmm* expression, by observing the GFP signal in the salivary gland. Furthermore, we evaluated the effects of oral administration of histone deacetylase (HDAC) inhibitors and vegetable-powders on* bmm* expression using the transgenic fly.

## 2. Materials and Methods

### 2.1. Materials

NCC-149 (HDAC8 inhibitor) and T302 (an HDAC9 inhibitor) were provided by Professor Takayoshi Suzuki (Kyoto Prefectural University of Medicine, Kyoto, Japan) [13, 14]. The following edible portions of vegetables were provided by Designer Foods Co. Ltd. (Nagoya, Japan): leaves of spinach and komatsuna; leaf heads of cabbage and lettuce; leaves and bud/flower of nabana (*Brassica* flower), broccoli, and edible flower; bulbs of onion; fruits of red paprika and tomato; and roots of Japanese radish. These vegetables were lyophilized and ground in a mill before use. Mulberry leaves harvested in Kyotango city (Kyoto, Japan) were dried and ground by air flush at 180°C for 7 s.

### 2.2. Recombinant Plasmid Construction

DNA fragments containing the* bmm* promoter were used for checking the promoter activity. The 2 kbp fragment from −1655 to +345 with the expected transcription initiation site was amplified by PCR using genomic DNA of yellow white (*yw*)* Drosophila*. The primers* bmm*F −1655 and* bmm*R +345 containing* Not*I site (underlined) were employed:

 
*bmm*F −1655: 5′-ATCAGATCCGCGGCCGCTTGAAGTGATTGGTAGTGGGTG-3′.
 
*bmm*R +345: 5′-GCTCACCATGCGGCCGCGCTTTGGACTCGGCGTTAGATT-3′.

The fragment obtained was inserted into the* Not*I site of plasmid pOBP-GFP (in which GFP was inserted into the pOBP vector derived from transposable P-element) with the aid of the In-Fusion PCR Cloning Kit (Clontech, Mountain View, CA, USA) [15]. Thus, the plasmid pOBP-*bmm* promoter-GFP for generating transgenic flies was obtained. A recombinant plasmid carrying the p53 promoter region of* Drosophila* (pOBP-p53 promoter-GFP) was used as the positive control [15].

### 2.3. Transfection of* Drosophila* Cells

The recombinant vector was transfected into S2-DRSC* Drosophila* cells (*Drosophila* Genomics Resource Center, Bloomington, IN, USA) with HilyMax transfection reagent (Dojindo, Kumamoto, Japan). At 48 h after transfection, the cells were collected, fixed with paraformaldehyde, and then stained with 4′,6-diamidino-2-phenylindole (DAPI, Invitrogen, Carlsbad, CA, USA). The cells were mounted on glass slides using Vectashield Mounting Medium (Vector Laboratories Ltd.) and were observed for DAPI and GFP signals under a confocal laser scanning microscope (Model Fv10i, Olympus Corp., Tokyo, Japan).

### 2.4. Establishment of Transgenic Flies

According to previous studies, P-element-mediated germ-line transformation was carried out by microinjecting pOBP-*bmm* promoter-GFP into fertilized eggs. Based on rescue of the white eye color, F1 transformants (*yw*; +;* bmm* promoter-GFP) were selected [16]. Because the* bmm* promoter activities in several independent transgenic lines were almost the same, line 17 carrying the* bmm* promoter-GFP gene on the third chromosome was used for further experiments.

### 2.5. Glucose Diet Feeding

The standard food supplement contained 0.8% agar (w/v), 9% cornmeal (w/v), 4% dry yeast (w/v), 0.05% (w/v) ethyl* p*-hydroxybenzoate, and 0.5% propionic acid (v/v). For preparation of the glucose diet, glucose was added to the standard food supplement to a final concentration range of 2.5–20% (w/v). Five male and 5 female transgenic flies were mated and allowed to lay eggs on the glucose-supplemented diet for 2 days at 25°C. The hatched larvae were grown on the same diet. Then, the third-instar larvae were used for lipid measurement or were dissected to analyze the GFP signal as described below.

### 2.6. Image Analysis

The third-instar larvae were dissected in phosphate-buffered saline (PBS), and the tissues collected were fixed in 3% paraformaldehyde for 30 min at room temperature. The tissues were washed 3 times with PBS and then set onto the glass slides using the Vectashield Mounting Medium. Images were acquired at 589 nm excitation/510 nm emission under a fluorescence microscope (Model BX-50, Olympus) equipped with a cooled CCD camera (ORCA-ER, Hamamatsu Photonics K.K., Shizuoka, Japan). The intensity of the GFP signal from the nucleus of the salivary gland was analyzed by using the MetaMorph software (version 7.7.7.0, Molecular Devices, Sunnyvale, CA, USA). The average intensity was used for calculating the GFP signal after being normalized by the background intensity.

### 2.7. Measurement of Lipid Content

The lipid content in flies was measured by the colorimetric sulfophosphovanillin (SPV) method as described previously [17, 18]. Ten third-instar larvae were homogenized in 2% sodium sulfate, and then chloroform/methanol (1 : 1) was added. The supernatant was collected by centrifugation (1000 ×g, 1 min), mixed with distilled water, and centrifuged again (1000 ×g, 1 min). For lipid measurement, the chloroform layer was first dried at 90°C for 10 min, and then 98% sulfuric acid was added and the solution was incubated for 10 min at 90°C. After cooling to room temperature, SPV reagent was mixed into the solution and the color development was measured at 530 nm.

### 2.8. HDAC Inhibitors and Vegetable-Powder Feeding

HDAC inhibitors were dissolved in ethanol and then diluted with distilled water. The solution was mixed with instant medium Formula 4–24 (Carolina Biological Supply Co., Burlington, NC, USA) to obtain a final HDAC inhibitor concentration of 0.5% (w/w). The control medium was prepared by mixing instant medium Formula 4–24 and distilled water together with the same concentration of ethanol used to dissolve the HDAC inhibitors.

After lyophilization, the vegetable-powder was mixed with instant medium Formula 4–24 to obtain a final concentration of 10% (w/w). Water was mixed well into the powder mixture to constitute the vegetable diet. The instant medium dissolved with water was used as a control medium.

Five male and 5 female transgenic flies were mated on the diet containing HDAC inhibitors or various vegetable-powders for 2 days. Newborn larvae were continuously fed on the same mediums until they reached the third-instar larval stage at 25°C. The salivary glands of the third-instar larvae were used to analyze the GFP intensity as described above.

### 2.9. Quantitative RT-PCR

Total RNAs were extracted from the whole body of third-instar larvae of transgenic flies and analyzed by a LightCycler Nano System with SYBR Green (FastStart SYBR Green Master Mix, Roche Diagnostics Corp., Indianapolis, IN, USA), as described previously [19]. The* Rp49* gene was used as an endogenous reference gene. Primers used were as follows:

 
*Rp49*-F76: 5′-AATCTCCTTGCGCTTCTTGG-3′.
 
*Rp49*-R214: 5′-TTACGGATCGAACAAGCGC-3′.
 
*bmm*-F466: 5′-CTGCTGTCTCCTCTGCGATTT-3′.
 
*bmm*-R606: 5′-TTCTGTAGACCCTCCAGCAG-3′.

Experiments were performed in triplicate for each of the three RNA batches isolated separately. The results collected from the real-time PCR were analyzed by 2^−ΔΔ^ (Livak) method, based on the observation of fold changes in mRNA levels [20].

## 3. Results

### 3.1. Establishment of the Fly Model for* bmm* Expression Monitoring

**Figure 1:**
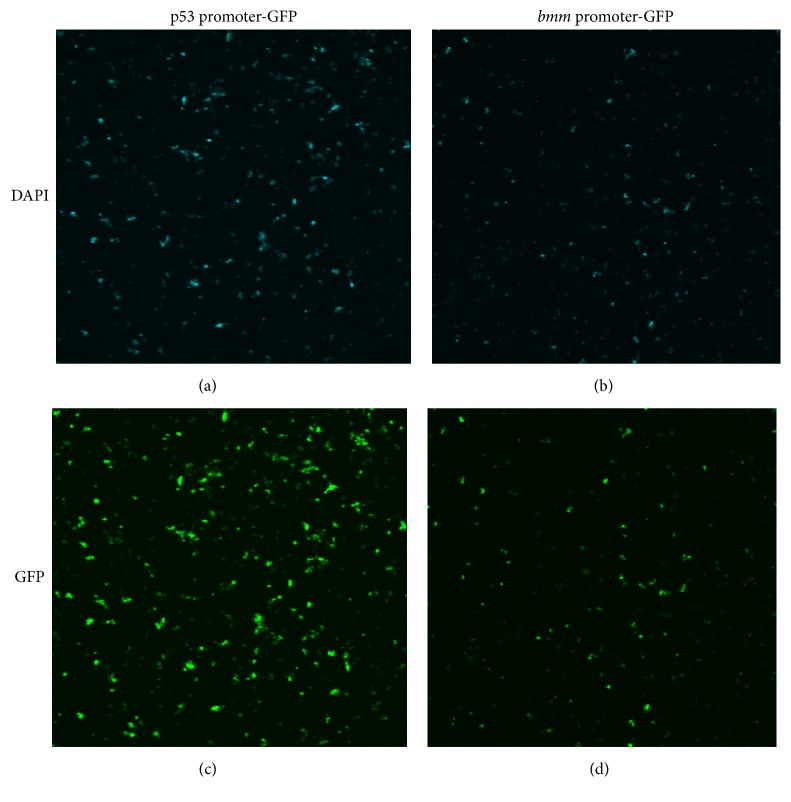
Transfection of S2-DRSC* Drosophila* cells. S2-DRSC* Drosophila* cells were transfected with pOBP-*bmm* promoter-GFP (b, d) or with pOBP-p53 promoter-GFP as a positive control (a, c). After staining with DAPI, cells were observed for DAPI signals (a, b) and GFP signals (c, d) under a fluorescence microscope. Cells transfected with pOBP-*bmm* promoter-GFP showed GFP signals, indicating that the* bmm* promoter functioned as expected.

We constructed a pOBP-*bmm* promoter-GFP plasmid carrying the 2 kbp 5′-flanking region of the* bmm* gene. To evaluate the* bmm* promoter activity, the plasmid was transfected into* Drosophila* S2-DRSC cells and the GFP signals were inspected by fluorescence microscopy. The cells transfected with pOBP-*bmm* promoter-GFP showed the GFP signal (Figure 1(c)), similar to the cells transfected with pOBP-p53 promoter-GFP as a positive control (Figure 1(d)). The results indicated that the 5′-flanking region of* bmm* amplified in this study was functional for promoter activity. Therefore, pOBP-*bmm* promoter-GFP was used to establish the transgenic flies.

**Figure 2:**
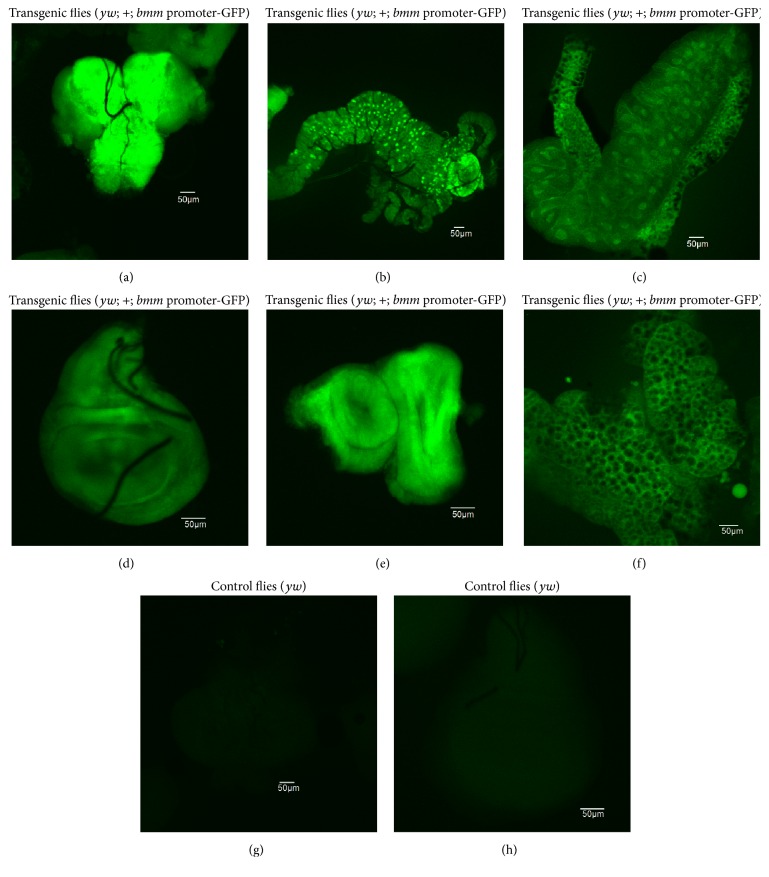
The* bmm* promoter-driven expression of GFP in transgenic* Drosophila* (*yw*; +;* bmm* promoter-GFP). The various tissues of the third-instar larvae of transgenic flies were observed by fluorescence microscopy. The sections of brain lobe (a), gut (b), salivary gland (c), wing disc (d), eye disc (e), and lipid tissue (f) showed GFP signals. In contrast, the control fly (*yw*) showed no detectable GFP signal ((g) brain lobe; (h) wing disc).

*D. melanogaster* was transformed with pOBP-*bmm* promoter-GFP and then the GFP expression in the third-instar larvae was analyzed. Under the fluorescence microscope, all observed tissues such as brain lobe, gut, salivary gland, wing disc, eye disc, and lipid tissue of transgenic flies displayed the green fluorescence of GFP (Figures 2(a)–2(f)). In contrast, there was no detectable GFP signal in the brain lobe (Figure 2(g)) and wing disc (Figure 2(h)) of control flies, indicating that the* bmm* promoter had functioned as expected in live flies.

### 3.2. Relationship between Lipid Storage and GFP Intensity

**Figure 3:**
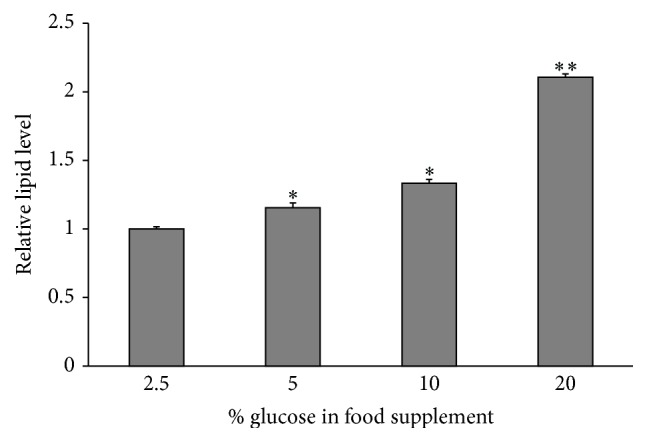
Relative lipid level in transgenic flies fed different glucose diets. The lipid level was measured by the sulfophosphovanillin method and normalized with body weight. The lipid content increased with increasing glucose content in the food supplements. *n* = 30 for each treatment. The error bars represent the standard deviation. ^*∗*^ *p* < 0.05; ^*∗∗*^ *p* < 0.001.

In order to check the usefulness of the transgenic fly model in evaluating obesity and screening antiobesity agents, the relationship between* bmm* expression and lipid content in flies was analyzed. First, we measured the lipid content of flies that had been orally given different concentrations of glucose (2.5–20%). As shown in Figure 3, the third-instar larvae fed higher glucose diets showed significantly increased lipid storage, whereas flies fed lower glucose diets had lower lipid content. This indicated that* Drosophila* on higher glucose diets had become obese, whereas those on lower glucose diets remained thin.

**Figure 4:**
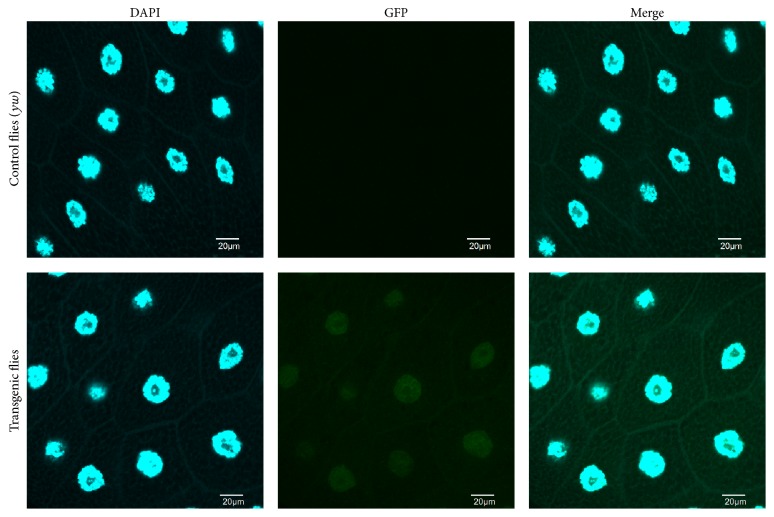
Intracellular localization of the GFP signal in the salivary gland. After staining with DAPI, the salivary glands of the third-instar larvae were observed by fluorescence microscopy. There was no detectable GFP signal in control flies (*yw*), whereas GFP carrying the nuclear localization sequence was detected in the nucleus of transgenic* Drosophila* (*yw*; +;* bmm* promoter-GFP).

**Figure 5:**
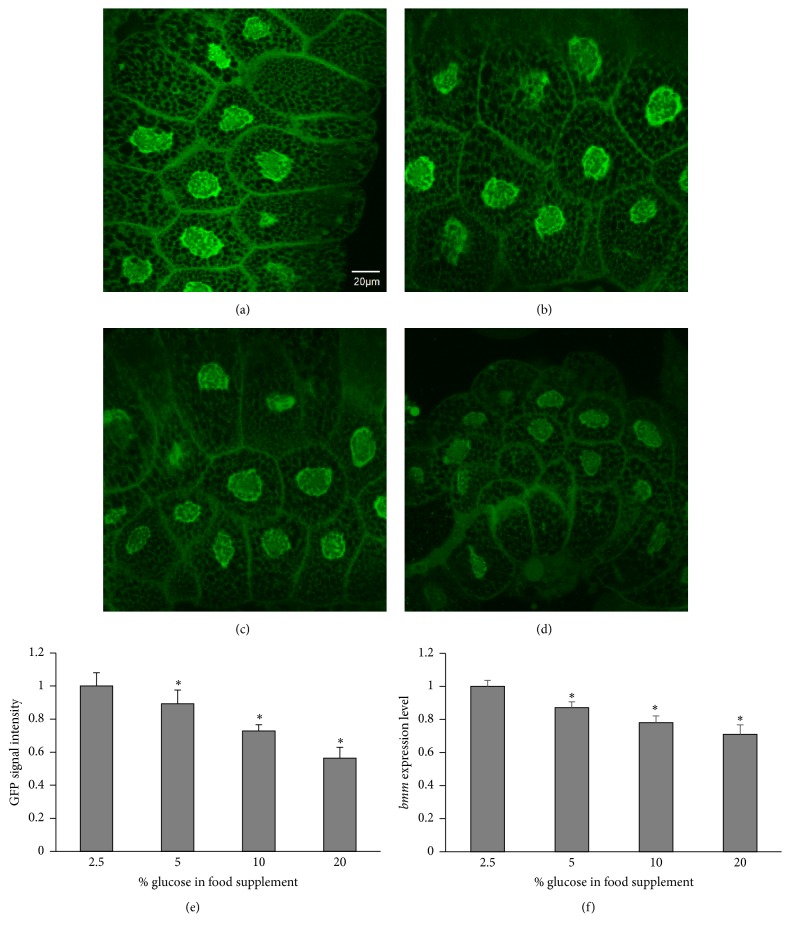
Relationship between* bmm* expression and glucose contents in diets. The transgenic flies were fed with diet containing different amounts of glucose, and the salivary glands of the third-instar larvae were observed by fluorescence microscopy. GFP signal intensities measured by the MetaMorph software (e) showed that flies fed 2.5% glucose (a) showed stronger signals than flies fed 5% (b), 10% (c), and 20% (d) glucose, respectively. Real-time PCRs were used to measure the endogenous* bmm* mRNA expression levels of transgenic flies (f). *n* = 30 for each treatment. The error bars represent the standard deviation. ^*∗*^ *p* < 0.05.

The GFP expression driven by the* bmm* promoter was localized to the nucleus of transgenic flies, as shown in Figure 4, because it contained the nucleus localization signal sequence [15]. Since the nucleus of the* Drosophila* salivary gland is very large as a result of endoreplication, the salivary gland of flies fed a glucose diet was used to detect the GFP signal by fluorescence microscopy (Figures 5(a)–5(d)). The intensity of the GFP signal decreased with increasing dietary glucose concentration (Figure 5(e)). Furthermore, quantitative RT-PCR analysis of the mRNA expression of* bmm* in the whole body of transgenic flies found the levels to be reduced upon increasing glucose amount in the diet (Figure 5(f)). These results correlated well with a previous report that had demonstrated the upregulation of* bmm* expression upon starvation and its downregulation upon refeeding [7], suggesting that this transgenic fly can be used for screening antiobesity drug or food candidates.

### 3.3. HDAC Inhibitors as Potential Antiobesity Agents

**Figure 6:**
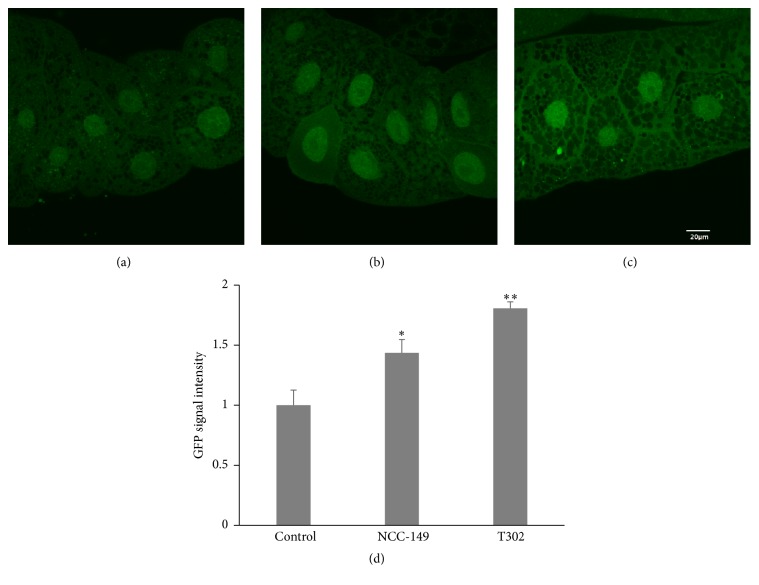
Effects of oral administration of histone deacetylase (HDAC) inhibitors on* bmm* expression in* Drosophila*. The transgenic flies were fed a diet containing HDAC inhibitors, and the salivary glands of the third-instar larvae were observed by fluorescence microscopy to evaluate* bmm* promoter-driven GFP signaling as a marker. GFP signals of* Drosophila* fed with instant food alone (a), instant food with NCC-149 (HDAC8 inhibitor) (b), or instant food with T302 (an HDAC9 inhibitor) (c). The GFP intensity measured by the MetaMorph software was enhanced in flies fed the HDAC inhibitors, and significant differences were observed relative to the control (d). *n* = 20 for each treatment. The error bars represent the standard deviation. ^*∗*^ *p* < 0.05; ^*∗∗*^ *p* < 0.001.

To evaluate the effects of HDAC inhibitors on* bmm *expression, we fed transgenic flies with 0.5% (w/w) of NCC-149 (HDAC8 inhibitor) or T302 (an HDAC9 inhibitor). As shown in Figure 6, the GFP intensities increased 1.4-fold and 1.8-fold relative to the control after administration of NCC-149 and T302, respectively. These results indicated that HDAC inhibitors can potentially be new antiobesity drugs.

### 3.4. Effect of Vegetables on* bmm* Expression

*Drosophila* that had been orally fed with nabana, spinach, lettuce, red paprika, cabbage, broccoli, Japanese radish, edible flower, or mulberry leaf showed increased GFP intensity in the salivary gland (Table 1). This suggests that these vegetables may suppress obesity. In contrast, flies fed with tomato showed a 0.74-fold decrease in the GFP signal relative to control flies, indicating enhanced lipid storage, whereas flies fed with komatsuna or onion showed no difference in GFP signal strength from that of control. Taken together, our screening identified several vegetables that have potential for preventing lipid accumulation in* Drosophila*.

## 4. Discussion

The development of a simple and rapid screening system using a living organism is important to screen new antiobesity substances. In the present study, the transgenic* Drosophila* fly carrying the fused genes of the* bmm* promoter and* GFP* showed fluorescence in all examined tissues, including salivary gland nuclei (Figure 2). In the past few years, GFP has been broadly used as a marker in fat storage studies in* C. elegans*. For instance, GFP was used as an indicator for RNAi screening to identify uncharacterized fat storage regulatory genes in* C. elegans* [12], and GFP fused with ATGL (*C. elegans* ortholog and a key lipolytic enzyme) was used to mark LDs in* C. elegans* [21]. GFP-fusion proteins are also widely applied for analyzing gene expression and protein localization in various* Drosophila* cell lines [5, 22]. The transgenic* bmm*-expressing* Drosophila* model established in this study has many advantages. First, the easy detection of the GFP signal due to the large size of the salivary gland nucleus enables the efficient screening for antiobesity candidate drugs. Second, fixing and staining of salivary gland tissue are not needed. Third, the GFP intensity in the salivary gland nucleus can be quantified easily by the MetaMorph software.

Obesity is associated with elevated levels of lipid content in tissues. Animals fed low- or high-calorie food can experience a decrease or an increase of their lipid level, respectively. In this study, we designed an experiment to culture* Drosophila* under different food conditions that could affect their lipid storage, while keeping the other compositions constant. By using the SPV method to measure total lipids, we proved that the lipid content in the fly body was elevated with increasing glucose content in the food diet. The transgenic* bmm*-expressing flies on low-glucose diets showed enhanced GFP signals; conversely, flies fed on high-glucose diets showed significantly decreased GFP signal intensities, indicating enhanced lipid storage. These results suggest that the expression of* bmm* is upregulated with low-calorie foods and downregulated with high-calorie foods, in good concordance with previous reports [7]. The inverse correlation between lipid level and the GFP intensity indicates that this transgenic fly could be a useful model for screening novel antiobesity drug or food candidates.

The present study indicates that HDAC inhibitors might be remarkable candidates for further obesity-therapy studies. In addition, our finding that those vegetables are potential to prevent obesity coincided well to previous report [23]. By adding 0.5% (w/w) HDAC8 and HDAC9 inhibitors to the culture medium, the GFP intensity in the transgenic* bmm*-expressing flies increased significantly compared with the control, indicating that obesity was suppressed. In previous studies, HDAC9 was proposed as a potential therapeutic target for obesity, since lipid accumulation was prevented in HDAC9-knockdown mice fed chronic high-fat diets [24]. In contrast, the potential of HDAC8 inhibitors for diabetes treatment has not yet been reported. Our results indicate that HDAC inhibitors not only of HDAC9 but also of HDAC8 may be new potential candidates as antiobesity drugs. In addition, when the transgenic* bmm*-expressing flies were fed with vegetable-powder-containing food, some of the vegetables induced the GFP signal, suggesting a reduction in lipid storage. Interestingly, cabbage and broccoli, which showed potential for preventing obesity in this study, both contain sulforaphane [25], a compound that has been reported to inhibit HDAC activity [26, 27]. Other groups have reported that tomato contains 9-oxo-10(E),12(E)-octadecadienoic acid and its derivatives, which are potent PPAR*α* agonists that decrease triglyceride accumulation in mouse primary hepatocytes [28, 29]. In contrast, our data suggested that tomato may induce lipid accumulation in* Drosophila*. The possible reason for this discrepancy is that the glucose and fructose in tomato can suppress* bmm* expression.

## 5. Conclusions

We have developed a* Drosophila* model with GFP indicating ability to be used as a lipid storage marker. Our model is recommended for the fast and simultaneous screening of a large number of samples. In addition, because of the similarities of the basic metabolic functions and analogous organs between* Drosophila* and vertebrates [5], this* Drosophila bmm*-expressing monitor constructed in our study is a promising model for the screening of novel drugs for treating obesity.

## Figures and Tables

**Table 1:** Effects of oral administration of vegetables on *bmm* expression in *Drosophila*.

Vegetable		Relative GFP intensity
Nabana	*Brassica napus* L.	1.49^*∗∗*^ ± 0.07
Spinach	*Spinacia oleracea* L.	1.33^*∗*^ ± 0.12
Lettuce	*Lactuca sativa*	1.79^*∗∗*^ ± 0.10
Red paprika	*Capsicum annuum* L.	1.92^*∗∗*^ ± 0.13
Cabbage	*Brassica oleracea* L. var. *capitata*	2.18^*∗∗*^ ± 0.31
Tomato	*Solanum lycopersicum* L.	0.74^*∗*^ ± 0.07
Komatsuna	*Brassica rapa* L. var. *perviridis* cv. Komatsuna	1.11^ns^ ± 0.22
Onion	*Allium cepa* L.	1.16^ns^ ± 0.23
Broccoli	*Brassica oleracea* L.	1.60^*∗∗*^ ± 0.11
Japanese radish	*Raphanus sativus* L.	1.44^*∗∗*^ ± 0.32
Edible flower	*Brassica rapa* L. var. *nippo-oleifera*	1.83^*∗∗*^ ± 0.25
Mulberry	*Morus alba* L.	2.25^*∗∗*^ ± 0.19

The transgenic flies were fed with a vegetable diet, and green fluorescent protein (GFP) signals in the salivary gland nuclei of the third-instar larvae were detected. The GFP intensity was analyzed using the MetaMorph software and was subtracted by that of the background signal; then the GFP intensity of the vegetable group relative to that of the control group was calculated. *n* = 6 for each sample. ^*∗*^ *p* < 0.05. ^*∗∗*^ *p* < 0.01. ^ns^Not significant.
